# Supplementary figures and images for: Unlocking epitope similarity: A comparative analysis of the American manatee (Trichechus manatus) IgA and human IgA through an immuno-informatics approach
Source: PLoS One. 2024 Sep 16;19(9):e0308396. doi: 10.1371/journal.pone.0308396 (PMC11404806; doi:10.1371/journal.pone.0308396)

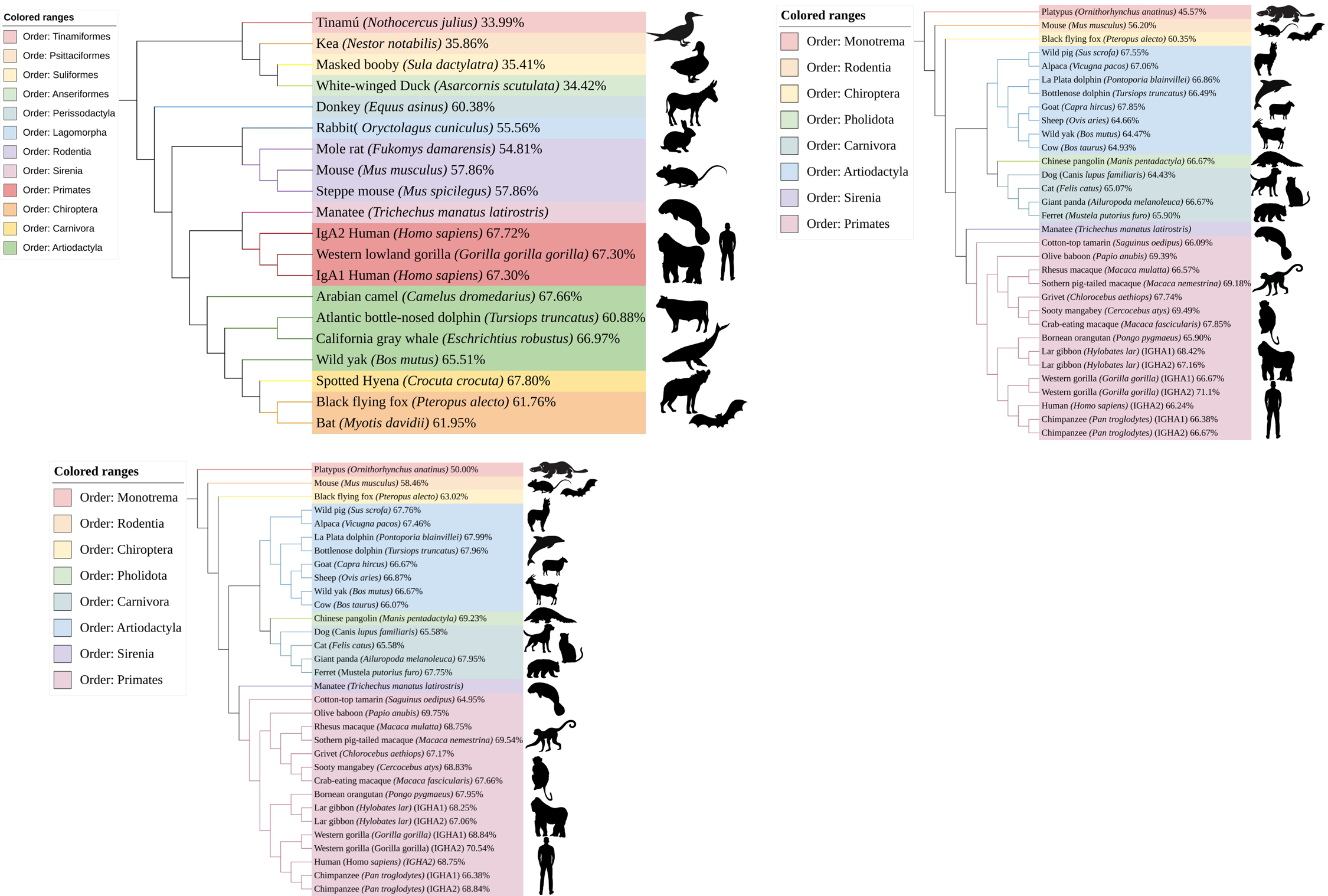

Supplement: S1 Fig — A: Tree generated from the complete IgA sequences using the hits from Uniprot. B: Tree of the complete IgA sequence using the hits from NCBI. C: Tree of the constant regions of IgA (CH1, CH2, CH3) using the hits from NCBI. (TIF) [file pone.0308396.s001.tif]

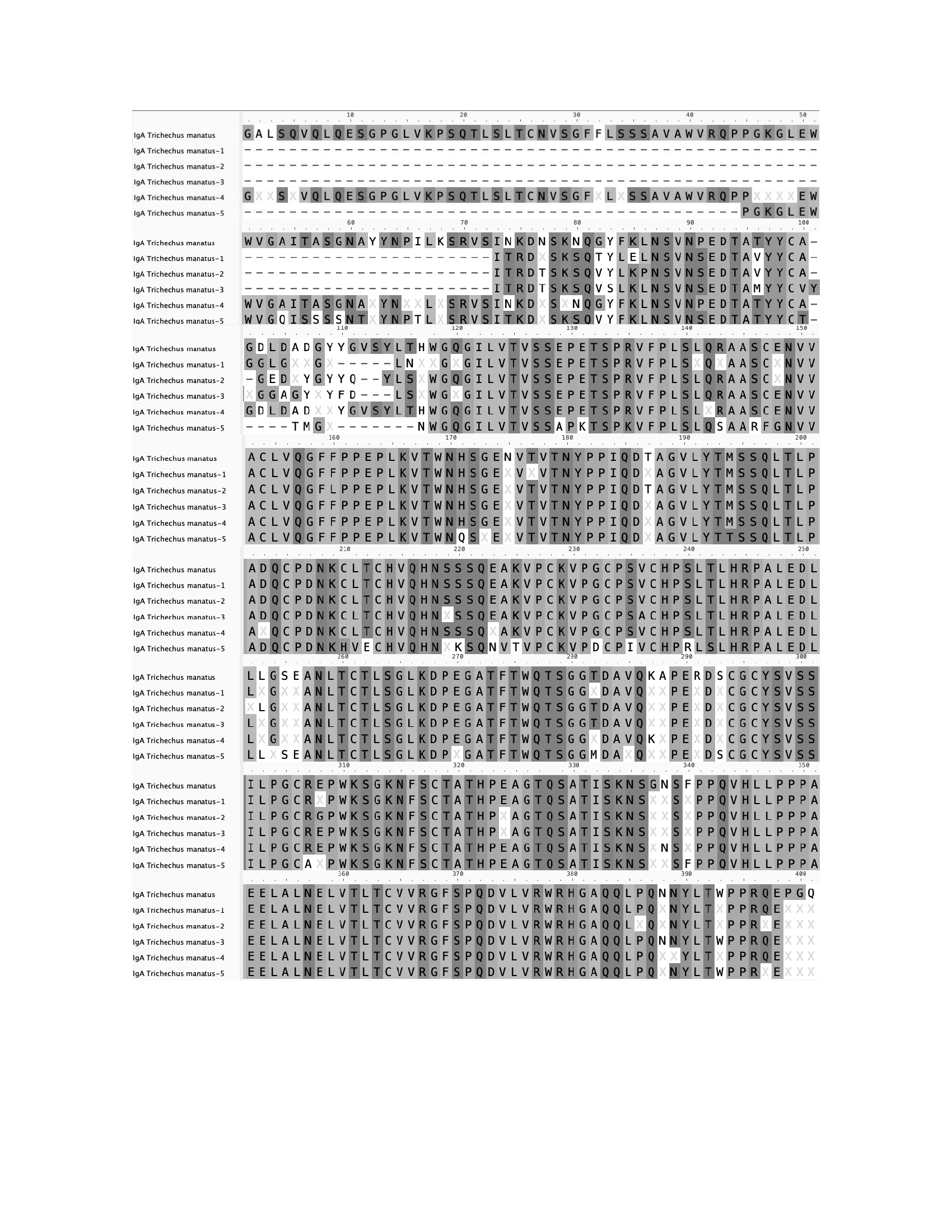

Supplement: S2 Fig — X represent amino acids that passes the detection threshold during the prediction. (TIF) [file pone.0308396.s002.tif]
