# Supplementary material for: Unlocking epitope similarity: A comparative analysis of the American manatee (Trichechus manatus) IgA and human IgA through an immuno-informatics approach
Source: PLoS One. 2024 Sep 16;19(9):e0308396. doi: 10.1371/journal.pone.0308396 (PMC11404806; doi:10.1371/journal.pone.0308396)
Supplement: S1 File — S1 Table. NCBI and UniProt hits of complete IgA. Accession number, species name, sequence name and identity percentage for each hit. S2 Table. NCBI and UniProt hits of constant regions (CH1, CH2, CH3) of IgA. Accession number, species name, sequence name and identity percentage for each hit. S3 Table. Consensus Linear B-cell Epitopes and Scores. S4 Table. Example of predicted manatee epitope and confirmed human epitope comparison. ID of the epitope sequence on the IEDB data base; Shared amino acids between the predicted epitope on the manatee IgA sequence with the confirmed epitopes in the IDEB database; Position over the reference sequence of human IgA1. (PDF) [file pone.0308396.s003.pdf]

S1 Table. NCBI and UniProt hits of complete IgA with accesion number, species name, sequence name and identity percentage

BLASTp results NCBI			
ID	Species	Sequence name	Identity percentage
AQR55609.1	*Trichechus manatus latitrostris*	IgA partial	-
CAA37744.1	*Gorilla gorilla*	immunoglobulin alpha-2 heavy chain, partial	71.1%
AAX73304.1	*Ailuropoda melanoleuca*	immunoglobulin alpha heavy chain, partial	66.67%
KAI5162510.1	*Manis pentadactyla*	Immunoglobulin Heavy Constant Alpha 1	66.67%
AAT65195.1	*Tursiops truncatus*	immunoglobulin IgA heavy chain constant region, partial	66.49%
P0DOX2.2	*Homo sapiens*	Immunoglobulin alpha-2 heavy chain;	66.24%
ATI97333.1	*Felis catus*	IgA constant region, partial	65.07%
AAC64980.1	*Ovis aries*	immunoglobulin alpha heavy chain, partial	64.66%
ADD71719.1	*Pteropus alecto*	immunoglobulin alpha heavy chain	60.35%
CAA37745.1	*Hylobates lar*	immunoglobulin alpha-1 heavy chain, partial	68.42%
CAO79580.1	*Vicugna pacos*	immunoglobulin heavy chain constant region of IgA secreted form, partial	67.06%
ATV90895.1	*Macaca mulatta*	immunoglobulin heavy chain alpha constant region, partial	66.57%
CAA37746.1	*Hylobates lar*	Immunoglobulin alpha-2heavy chain constant region, partial	67.16%
CAA33147.1	*Gorilla gorilla*	Ig alpha-1, partial	66.67%
AMP34155.1	*Capra hircus*	immunoglobulin alpha heavy chain, partial	67.85%
ABI97146.1	*Macaca nemestrina*	immunoglobulin alpha heavy chain constant region, partial	69.18%
CAA37742.1	*Macaca fascicularis*	immunoglobulin alpha heavy chain, partial	67.85%
PNI15543.1	*Pan troglodytes*	IGHA1 isoform 2, partial	66.67%
NIG60332.1	*Pontoporia blainvillei*	immunoglobulin IgA heavy chain constant region	66.86%
CAA37741.1	*Pongo pygmaeus*	immunglobulin alpha heavy chain, partial	65.90%
ADD51207.1	*Sus scrofa*	immunoglobulin IgA heavy chain constant region, partial	67.55%
CAA37739.1	*Pan troglodytes*	Immunoglobulin alpha-1 heavy chain, partial	66.38%
BAL37291.1	*Mus musculus*	immunoglobulin alpha chain	56.20%
QJY40729.1	*Mustela putorius furo*	IgA immunoglobulin constant region, partial	65.90%
WEL32157.1	*Chlorocebus aethiops*	immunoglobulin heavy constant alpha, secreted form, partial	67.74%
KAK2104939.1	*Saguinus oedipus*	Ig alpha-1 chain C region	66.09%
ABI97142.1	*Papio anubis*	immunoglobulin alpha heavy chain constant region, partial	69.39%
AAT11506.1	*Cercocebus atys*	immunoglobulin alpha heavy chain, partial	69.49%
ELR44852.1	*Bos mutus*	Ig alpha-1 chain C region, partial	64.47%
AAC98391.1	*Bos taurus*	immunoglobulin IgA heavy chain constant region, partial	64.93%
AAA56796.1	*Canis lupus familiaris*	IgA heavy chain constant region, partial	64.43%
AAL17700.1	*Ornithorhynchus anatinus*	IgA1 immunoglobulin	45.57%

BLASTp results UniProt			
ID	Species	Sequence name	Identity percentage
P01877-1	*Homo sapiens*	Isoform 1 of Immunoglobulin heavy constant alpha 2	69.73%
P20758	*Gorilla gorilla gorilla*	Ig alpha-1 chain C region	69.82%
P01876-1	*Homo sapiens*	Isoform 1 of Immunoglobulin heavy constant alpha 1	69.82%
L8HP94	*Bos mutus*	Ig alpha-1 chain C region	67.62%
L5JPI9	*Pteropus alecto*	Ig alpha-1 chain C region	51.31%
L5MCH2	*Myotis davidii*	Ig alpha-1 chain C region	62.67%
A0A6J3R1N9	*Tursiops truncatus*	Immunoglobulin heavy constant alpha 2	58.62%
P01878	*Mus musculus*	Ig alpha-1 chain C region	58.75%
A0A8C6MR51	*Mus spicilegus*	Immunoglobulin heavy constant alpha	57.73%
A0A5N4E0V1	*Camelus dromedarius*	Ig alpha-1 chain C region	69.55%
P0DUB3	*Equus asinus*	Immunoglobulin heavy constant alpha	61.68%
A0A091EIJ7	*Fukomys damarensis*	Ig alpha-1 chain C region	56.33%
A0A6G1BA63	*Crocuta crocuta*	IGHA1 protein	63.38%
A0A2F0BNF0	*Eschrichtius robustus*	Ig alpha-1 chain C region	62.97%
P01879	*Oryctolagus cuniculus*	Ig alpha-1 chain C region	56.58%
A0A7K7LCA2	*Asarcornis scutulata*	IGHA2 protein	35.98%
A0A7K7WVI2	*Nothocercus julius*	IGHA2 protein	35.67%
A0A091RUN3	*Nestor notabilis*	Ig alpha-1 chain C region	38.39%
A0A851APM2	*Sula dactylatra*	IGHA1 protein	37.00%
A0A1S6EEL0	*Trichechus manatus latitrostris*	IgA	-

S2 Table. NCBI and UniProt hits of constan regions (CH1, CH2, CH3) of IgA with accesion number, species name, and identity percentage

Sequences retrived from NCBI		
CH1, CH2, CH3		
ID	Species	Identity percentage
AQR55609.1	*Trichechus manatus latitrostris*	-
CAA37744.1	*Gorilla gorilla*	70.54%
AAX73304.1	*Ailuropoda melanoleuca*	67.95%
KAI5162510.1	*Manis pentadactyla*	69.23%
AAT65195.1	*Tursiops truncatus*	67.96%
P0DOX2.2	***Homo sapiens***	68.75%
ATI97333.1	*Felis catus*	65.58%
AAC64980.1	*Ovis aries*	66.87%
ADD71719.1	*Pteropus alecto*	63.02%
CAA37745.1	*Hylobates lar*	68.25%
CAO79580.1	*Vicugna pacos*	67.46%
ATV90895.1	*Macaca mulatta*	68.75%
CAA37746.1	*Hylobates lar*	67.06%
CAA33147.1	*Gorilla gorilla*	68.84%
AMP34155.1	*Capra hircus*	66.67%
ABI97146.1	*Macaca nemestrina*	69.54%
CAA37742.1	*Macaca fascicularis*	67.66%
PNI15543.1	*Pan troglodytes*	68.84%
NIG60332.1	*Pontoporia blainvillei*	67.99%
CAA37741.1	*Pongo pygmaeus*	67.95%
ADD51207.1	*Sus scrofa*	67.76%
CAA37739.1	*Pan troglodytes*	69.14%
BAL37291.1	*Mus musculus*	58.46%
QJY40729.1	*Mustela putorius furo*	67.75%
WEL32157.1	*Chlorocebus aethiops*	67.17%
KAK2104939.1	*Saguinus oedipus*	64.95%
ABI97142.1	*Papio anubis*	69.75%
AAT11506.1	*Cercocebus atys*	68.83%
ELR44852.1	*Bos mutus*	66.67%
AAC98391.1	*Bos taurus*	66.07%
AAA56796.1	*Canis lupus familiaris*	65.58%
AAL17700.1	*Ornithorhynchus anatinus*	50.00%

Sequences retrived from UniProt		
CH1, CH2, CH3		
ID	Species	Identity percentage
P01877-1	***Homo sapiens (IGHA2)***	**67.72%**
P20758	*Gorilla gorilla gorilla*	67.30%
P01876-1	***Homo sapiens (IGHA1)***	**67.30%**
L8HP94	*Bos mutus*	65.51%
L5JPI9	*Pteropus alecto*	61.76%
L5MCH2	*Myotis davidii*	61.95%
A0A6J3R1N9	*Tursiops truncatus*	60.88%
P01878	· *Mus musculus*	57.86%
A0A8C6MR51	*Mus spicilegus*	57.86%
A0A5N4E0V1	*Camelus dromedarius*	67.66%
P0DUB3	*Equus asinus*	60.38%
A0A091EIJ7	*Fukomys damarensis*	54.81%
A0A6G1BA63	*Crocuta crocuta*	67.80%
A0A2F0BNF0	*Eschrichtius robustus*	66.97%
P01879	*Oryctolagus cuniculus*	55.56%
A0A7K7LCA2	*Asarcornis scutulata*	34.42%
A0A7K7WVI2	*Nothocercus julius*	33.99%
A0A091RUN3	*Nestor notabilis*	35.86%
A0A851APM2	*Sula dactylatra*	35.41%
A0A1S6EEL0	*Trichechus manatus latitrostris*	-

S3 Table. Consensus Linear B-cell Epitopes and Scores

		IEBD		BcePred							ABCpred
Epitope	Domain	Bepipred-1.0	Bepipred-2.0	BCPREDHY	BCRED FLEX	BCPRED ACC	BCPRED TURNS	BCPRED EXP	BCPRED POL	BCPRED ANT	ABCPRED
DTSKS	VH	0,870733333	0,529421667	2,53075	2,760225	2,32232619	-	2,088097253	-	-	0,786
NSVNSEDT	VH	0,925519445	0,536493333	2,29945	2,237833333	-	2,156375	-	-	-	0,795
LVTVSSEPETSPRVFP	CH1	1,324795556	0,597177119	2,368	2,222	2,28575	-	-	2,25884	2,18823	0,88
WNHSGENVTV	CH1	1,32479556	0,567047021	-	-	-	2,345666667	-	-	-	0,804
DQCPDN	CH1	0,85162	0,531686667	2,262	-	2,1612	-	-	-	2,338157143	0,89
HNSSSQEAKVP	CH1	0,811537727	0,587919138	2,497	2,50356	2,177733333	2,95829	-	-	2,473441587	0,798
APERDS	CH2	1,150358621	0,5459375	2,1948	2,1855	2,501066667	-	-	-	-	0
EPWKSGNK	CH2	1,076577261	0,543185714		2,205333333	2,2228	-	-	-	-	0,848
GTQSATISKNSGN	CH2	1,076577261	0,59257239	2,159	2,512	-	2,3815	-	-	-	0,87
HLLPPPAEELALNE	CH3	0,8036	0,59257239	-	-	-	-	-	-	-	0,7
QLPQNN	CH3	0,763	0,58095	-	-	2,259333333	2,321	-	-	-	0,83
PRQEPG	CH3	1,449214286	-	2,260333333	2,016	2,387714286	-	-	-	-	-
QTWKWGD	CH3	0,827985714	0,555966667		-	2,211	-	-	-	-	0,89

S4 Table. Example of predicted manatee epitope and confirmed human epitope

**Sequence ID**																								**Shared amino acids**
Predicted epitope manatee IgA	-	-	-	-	-	-	-	-	-	**H**	**L**	**L**	**P**	**P**	**P**	**A**	**E**	**L**	**L**	-	-	-	-	10/10
939259	-	-	-	-	-	-	-	-	-	-	-	L	P	P	P	S	E	E	L	A	L	-	-	7/10
716125	-	-	-	-	-	-	-	-	-	-	L	L	P	P	P	S	E	E	L	-	-	-	-	8/10
730540	-	-	-	-	-	-	-	-	V	H	L	L	P	P	P	S	E	E	L	-	-	-	-	9/10
1286220	-	-	-	T	F	R	P	E	V	H	L	L	P	P	P	S	E	E	L	A	L	-	-	9/10
1277028	S	G	N	T	F	R	P	E	V	H	L	L	P	P	P	S	E	E	L	A	L	N	E	9/10
1223080	-	G	N	T	F	R	P	E	V	H	L	L	P	P	P	S	E	E		-	-	-	-	8/10
1277027	S	G	N	T	F	R	P	E	V	H	L	L	P	P	P	S	E	E	L	-	-	-	-	9/10
1277025	S	G	N	T	F	R	P	E	V	H	L	L	P	P	P	S	-	-	-	-	-	-	-	6/10
1048949	S	G	N	T	F	R	P	E	V	H	-	-	-	-	-	-	-	-	-	-	-	-	-	1/10
**IGHA1 Human position**	**222**	**223**	**224**	**225**	**226**	**227**	**228**	**229**	**230**	**231**	**232**	**233**	**234**	**235**	**236**	**237**	**238**	**239**	**240**	**241**	**242**	**243**	**244**	

ID of the epitope sequence on the IEDB data base; Shared amino acids between the predicted epitope on the Manatee IgA sequence with the confirmed epitopes in the IDEB database; Position over the reference sequence of human IgA1.
