# Supplementary material for: Unlocking epitope similarity: A comparative analysis of the American manatee (Trichechus manatus) IgA and human IgA through an immuno-informatics approach
Source: PLoS One. 2024 Sep 16;19(9):e0308396. doi: 10.1371/journal.pone.0308396 (PMC11404806; doi:10.1371/journal.pone.0308396)
Supplement: S2 File — The analysis includes: 1) The presence of the epitope in the five available manatee IgA sequences; 2) The conservation level of each amino acid position within the predicted epitopes; 3) The average conservation score per algorithm for the conserved epitopes; and 4) An interquartile normalization strategy, showing the localization of each average epitope score within the interquartile range. (DOCX) [file pone.0308396.s004.docx]

**Supplementary Methods**

**2.6 Conservation, identity, and similarity assessment**

The interquartile scale was calculated for each of the algorithms used. For this purpose, the detection limit was taken as the lower limit of Q1 to ensure that only epitopes passing this limit were included. Additionally, the upper limit of the detection range for each algorithm was taken as the upper limit of Q4. To determine quartiles, this created range was divided into 4. Once the limits were established, the consensus scores of each epitope were assigned to the corresponding range and given a score. Figure 1 exemplifies the implemented scale.

For Bepipred-1.0 Linear Epitope Prediction and BepiPred-2.0: Sequential B-Cell Epitope Prediction, since the limits of the detection range were unknown, the maximum and minimum value presented by any amino acid was used as the upper limit of Q4 and lower limit of Q1.

|  | HLDR | DT | Range |  | Q1 | Q2 | Q3 | Q4 |
| --- | --- | --- | --- | --- | --- | --- | --- | --- |
| **Bepipred 1** | 2,5 | 0,35 | 2,15 |  | 0,8875 | 1,425 | 1,9625 | 2,5 |
| **Bepipred 2** | 0,727 | 0,5 | 0,227 |  | 0,55675 | 0,6135 | 0,67025 | 0,727 |
| **BcePred-HY** | 3 | 2 | 1 |  | 2,25 | 2,5 | 2,75 | 3 |
| **BcePred -FLEX** | 3 | 1,9 | 1,1 |  | 2,175 | 2,45 | 2,725 | 3 |
| **BcePred -ACC** | 3 | 2 | 1 |  | 2,25 | 2,5 | 2,75 | 3 |
| **BcePred -TURNS** | 3 | 1,9 | 1,1 |  | 2,175 | 2,45 | 2,725 | 3 |
| **BcePred -EXP** | 3 | 2,4 | 0,6 |  | 2,55 | 2,7 | 2,85 | 3 |
| **BcePred -POL** | 3 | 2,3 | 0,7 |  | 2,475 | 2,65 | 2,825 | 3 |
| **BcePred -ANT** | 3 | 1,8 | 1,2 |  | 2,1 | 2,4 | 2,7 | 3 |
| **ABCPred** | 1 | 0,51 | 0,49 |  | 0,6325 | 0,755 | 0,8775 | 1 |
|  |  |  |  | **Score** | 0,25 | 0,5 | 0,75 | 1 |
| HLRD: Higher limit detection range, DT: detection threshold | | | | | | | | |

For the predicted epitope comparison with the human IgA confirmed epitopes, a similarity proportion was computed based on the number of shared amino acids between the epitopes. The number of amino acids in the predicted epitope served as the maximum, and a comparison was conducted with the confirmed epitopes. Any inherent difference between the sequences was considered a mismatch. All epitopes were included if they shared at least one amino acid. A range was established, considering the lowest and highest proportions found within each epitope. Finally, the total number of confirmed epitopes that matched a predicted epitope was taken into account. (see Table S4)
