# Supplementary material for: Unlocking epitope similarity: A comparative analysis of the American manatee (Trichechus manatus) IgA and human IgA through an immuno-informatics approach
Source: PLoS One. 2024 Sep 16;19(9):e0308396. doi: 10.1371/journal.pone.0308396 (PMC11404806; doi:10.1371/journal.pone.0308396)
Supplement: Attachment — Submitted filename: review.pdf [file pone.0308396.s005.pdf]

Review of manuscript PONE-D-24-17846 "Unlocking epitope similarity: a comparative analysis of manatee (Trichechus manatus) IgA and human IgA through an immuno-informatics approach."

Authors conducted a computational analysis from published genomic sequences to compare manatee IgA with that of other species available in looking from overdue the problem of cross-reaction when using a non-species-specific reagent in studies of immunological development of nurtured calves. This preliminary study is significantly useful to design protocols in monitoring development of calves nurtured with non-maternal milks.

This study is a basic step and its well based on previous knowledge. It´s a shame that there isn´t sequences available from nearly related species, as other afrotherians like elephants, but authors use the available data in their analysis. Also, it would be great to include sequences from other manatee species or subspecies or comment of the authors about expected variability among them.

Methods, algorithms, and software used by author are well exposed in the main text and supporting materials. The manuscript is well organized and written clearly enough to be accessible to non-specialists. I found it clearly written and I think is a valuable step to further studies in immunology in manatees.
