# Supplementary material for: Unlocking epitope similarity: A comparative analysis of the American manatee (Trichechus manatus) IgA and human IgA through an immuno-informatics approach
Source: PLoS One. 2024 Sep 16;19(9):e0308396. doi: 10.1371/journal.pone.0308396 (PMC11404806; doi:10.1371/journal.pone.0308396)
Supplement: Attachment — Submitted filename: Response to Reviewers.pdf [file pone.0308396.s006.pdf]

Bogotá DC, Colombia; June 28, 2024

Karolina Goździewska-Harłajczuk
Academic Editor PLOS ONE

Re: PONE-D-24-17846 Response to Reviewers

Respected Academic Editor and Reviewers,

Thank you for the opportunity to revise and resubmit our manuscript titled **"Unlocking epitope similarity: a comparative analysis of the American manatee (*Trichechus manatus*) IgA and human IgA through an immuno-informatics approach."** Reviewer's insightful comments and suggestions have significantly enhanced the quality of our manuscript. We have carefully considered each of your comments, and the changes made are highlighted throughout the manuscript with track changes. Below, we provide detailed responses to each of the suggestions and comments.

Journal requirements:

1. Please ensure that your manuscript meets PLOS ONE's style requirements, including those for file naming.
   Response: We have thoroughly revised the manuscript to ensure it meets PLOS ONE's style requirements, including adhering to the appropriate file naming conventions.

2. Please note that PLOS ONE has specific guidelines on code sharing for submissions in which author-generated code underpins the findings in the manuscript. In these cases, we expect all author-generated code to be made available without restrictions upon publication of the work.
   Response: Since our research was conducted relying upon freely available software and did not involve generating author-specific code, there are no codes underpinning our findings to share.

3. Please note that funding information should not appear in any section or other areas of your manuscript. We will only publish funding information present in the Funding Statement section of the online submission form. Please remove any funding-related text from the manuscript.
   Response: We have removed all funding-related text from the manuscript as per your guidelines. This information will now only be included in the Funding Statement section of the online submission form.

Reviewer #1 comments

1. Title: include the vernacular name.
   Response: We have included the vernacular name "American manatee" in the title as recommended.

2. Short title: include "manatee" or vernacular name
   Response: We have included the vernacular name "American manatee" in the short title as "American manatee IgA cross reactivity."

3. If you use the vernacular name of the subspecies "Caribbean manatee", provide the scientific name of the subspecies (*Trichechus manatus manatus*). If you are going to use only Trichechus manatus, use the vernacular name American manatee or just manatee. The vernacular name of the subspecies

should be "Antillean manatee" or "Weste Indian manatee" or "Greater Caribbean manatee", but the "Geater" was not included in the name.
Response: We have opted to consistently use "American manatee" (*Trichechus manatus*) as both the vernacular and scientific name throughout the text. While "American manatee" is used in the titles and initial mentions in the abstract and introduction, we use "manatee" subsequently in the text to maintain clarity and consistency.

4. Abstract is subjective. Need to improve writing.
Response: We have revised the abstract to enhance clarity and objectivity, ensuring it effectively communicates the study's objectives and results. Additionally, the text has been reviewed by both AI ChatGPT and an anglophone style revisor for further refinement.

5. Reference "1" is a regional action plan. Use a reference with greater global information to inform distribution.
Response: We have considered your feedback and replaced the reference with a source that offers a broader global perspective on the species' distribution.

6. Material and methods need to come before results.
Response: We have restructured the manuscript accordingly, with the Methods section now preceding the Results.

7. Inform whether the milk supplied to the manatee is with or without lactose.
Response: We have addressed the presence of lactose in milk substitutes in the introduction, detailing its concentrations and implications for manatee health.

8. I believe that reference 7 is not adequate to talk about the nutritional issues of manatees in captivity.
Response: We have reviewed the references and selected more appropriate sources to discuss the nutritional issues of manatees in captivity. The necessary changes have been made accordingly.

9. I strongly recommend reviewing references for the information they provide before citing them.
Response: We have thoroughly reviewed the references with two independent reviewers and updated some of them based on the information provided in each article.

10. The text does not make it clear whether the information on conservation comes from the study site or the entire area where the species occurs, especially with regard to food and the problems caused in captivity.
Response: We have revised the manuscript to clarify that the conservation information pertains to the entire range where the species is found, as supported by the references cited.

11. It is necessary to check the "Submission Guidelines" item to adapt the manuscript to the journal's standards.
Response: Following the reviewer's 1 suggestion, we have carefully examined the 'Submission Guidelines' and made necessary adjustments to ensure that the manuscript conforms to the journal's standards.

12. Vernacular and scientific name formats need to be standardized throughout the text. A rigorous review is necessary. the vernacular name must be outside the parenthesis and the scientific name inside.
Response: We have standardized the format of vernacular and scientific names throughout the manuscript as per your suggestion, with the vernacular name outside parentheses and the scientific name inside.

13. I didn't understand why from the "Supplementary Methods" file, this information can be described in the text.
Response: Thank you for your observation. We considered that including the detailed information from the "Supplementary Methods" file could potentially overwhelm the reader's attention from the main Methods section. However, we aimed to ensure comprehensive coverage of the data analysis process.

14. The text presents important results, but it needs to undergo text organization, adoption of standards and adaptation of names, before a more appropriate evaluation. I request an initial adaptation and organization of the text, so that it can be evaluated again.
Response: Thank you for your comprehensive evaluation of our manuscript and for your valuable comments. We appreciate the suggestions for improving text organization, adopting standards, and ensuring name consistency. These changes have been meticulously implemented to align with journal requirements. We look forward to your reassessment of the revised manuscript.

Reviewer #2 comments

1. "Authors performed an inmune-informatic study to compare manate IgA with that of other species, as a base knowledge to design further studies on manate IgA, specially in manatee breast-milk. Authors used available sequences and use computational algorithms to compare different aspects of IgA from other species. I think it is a well designed study presented in a clear manuscript."
Response: Thank you sincerely for your positive assessment of our study and manuscript.

2. Considere to use "American manatee" for the species, as a recent exercise from many specialists propose it in Mignucci et al. [in press Cariben Naturalist].
Response: Thank you for your suggestion regarding the vernacular name. We have opted to consistently use "American manatee" throughout the article, as recommended by specialists (Mignucci et al., in press, Caribbean Naturalist).

3. I think, author used the only species which have available sequences of IgA in public databases, but is not clear in the methods if this is true or if they took some species. In this sense, I wonder if there is IgA data on other afrotherians, like elephants or among other manatee species or subspecies.
Response: Thank you for raising this point. To address the study's focus on homology comparison, we specifically limited our analysis to the top 100 results in BLASTp, as detailed in the manuscript. Furthermore, we confirmed that IgA sequences from other Afrotheria species or additional manatee species are currently unavailable and were therefore not included in our study. These clarifications have been incorporated into the revised manuscript.

4. In discussion, several times authors repeat the statement about the finding of more similarity with human IgA, I found it some kind redundant.
Response: Thank you for highlighting this issue. We appreciate your feedback and have revised the discussion section accordingly to eliminate redundancy and improve clarity.

We sincerely appreciate the insightful feedback provided by the reviewers. Their comments have been invaluable in refining our manuscript to meet the rigorous standards of PLOS ONE. We have diligently addressed each point raised, including standardizing the vernacular and scientific names, clarifying methods and results sections, and ensuring adherence to submission guidelines. These revisions have strengthened the clarity, organization, and scientific rigor of our manuscript. We are confident that these enhancements significantly improve the manuscript and look forward to the reviewers' reassessment.

Thank you once again for the opportunity to improve our work and for your consideration. We hope that our manuscript will be given careful consideration for publication in PLOS ONE.

Sincerely yours,

AUGUSTO VALDERRAMA-AGUIRRE, M.SC. PH.D.
Assistant Professor, Department of Biological Sciences
Universidad de Los Andes, Bogotá DC, Colombia

Contact details: a.valderramaa@uniandes.edu.co, +57 318 329 9094
